# Operando Studies of Electrochemical Denitrogenation and Its Mitigation of N-Doped Carbon Catalysts in Alkaline Media

**DOI:** 10.1021/acscatal.2c05590

**Published:** 2023-02-09

**Authors:** Kai Zhao, Shihao Han, Le Ke, Xiaoyu Wu, Xiaoyu Yan, Xiaojuan Cao, Lingjiao Li, Xiaoyi Jiang, Zhiping Wang, Huijun Liu, Ning Yan

**Affiliations:** † School of Physics and Technology, Wuhan University, Wuhan 430072, China; ‡ Van’t Hoff Institute for Molecular Sciences, University of Amsterdam, Amsterdam 1098XH, The Netherlands

**Keywords:** denitrogenation, N-doped carbon corrosion, degradation mechanisms, corrosion inhibition, electrocatalysis

## Abstract

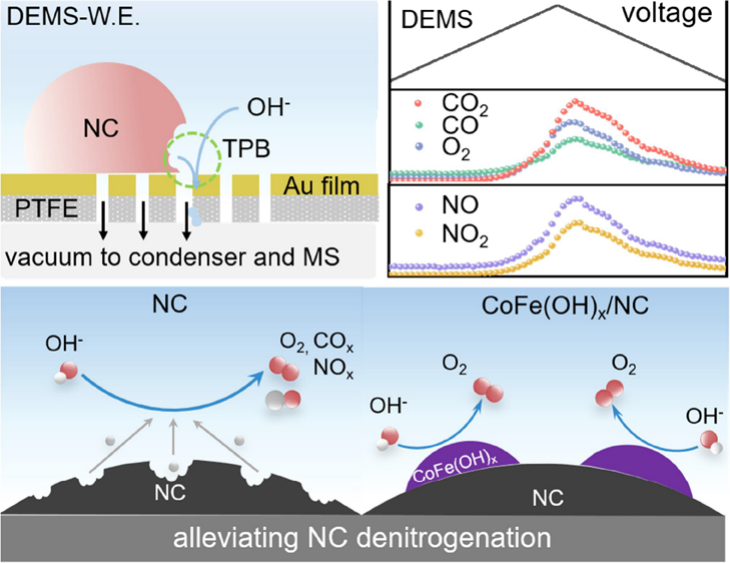

N-doped carbons (NCs) have excellent electrocatalytic performance in oxygen reduction reaction, particularly in alkaline conditions, showing great promise of replacing commercial Pt/C catalysts in fuel cells and metal–air batteries. However, NCs are vulnerable when biased at high potentials, which suffer from denitrogenation and carbon corrosion. Such material degradation drastically undermines the activity, yet its dynamic evolution in response to the applied potentials is challenging to examine experimentally. In this work, we used differential electrochemical mass spectroscopy coupled with an optimized cell and observed the dynamic behaviors of NCs under operando conditions in KOH electrolyte. The corrosion of carbon occurred at ca. 1.2 V vs RHE, which was >0.3 V below the measured onset potential of water oxidation. Denitrogenation proceeded in parallel with carbon corrosion, releasing both NO and NO_2_. Combined with the ex situ characterizations and density-functional theory calculations, we identified that the pyridinic nitrogen moieties were particularly in peril. Three denitrogenation pathways were also proposed. Finally, we demonstrated that transferring the oxidation reaction sites to the well-deposited metal hydroxide with optimized loading was effective in suppressing the N leaching. This work showed the dynamic evolution of NC under potential bias and might cast light on understanding and mitigating NC deactivation for practical applications.

## 1 Introduction

Replacing Pt-group metal (PGM)-containing catalysts is vital for the ubiquitous use of various emerging electrochemical energy conversion devices such as fuel cells^1−3^ and metal–air batteries.^4−6^ Over the past decade, heteroatom-doped carbons and their composite have been in the spotlight which demonstrate superior activity in oxygen reduction reactions (ORR)^7,8^ and oxygen evolution reactions (OER).^9,10^ Nitrogen-doped carbon (NC) is of particular interest in alkaline media: while its ORR activity is comparable with that of the commercial Pt/C catalyst,^11,12^ the similar atom radius and electronegativity between N and C atoms also enables its excellent thermodynamic stability. Besides, NC has been extensively studied as an ideal support to construct a composite electrocatalyst.^13,14^ The interaction between the nitrogen moieties and the supported nanoparticles offers an effective approach to optimizing the electronic and geometric structure for better catalysis.^15,16^

Carbon materials are vulnerable when biased at high potentials. Thermodynamically, carbon oxidation occurs at ca. 0.2 V versus a reversible hydrogen electrode (RHE).^17^ For instance, the charging process in metal–air batteries^6,18^ and the fuel starvation in fuel cells^17,19^ can easily induce the corrosion of carbon, forming gaseous, electrolyte-soluble, and -insoluble organic and inorganic products and causing electrocatalytic performance deterioration. Studying carbon corrosion under operando conditions offers the dynamic information of the materials in response to the potential bias and is fundamentally important to reveal the corrosion mechanism. NC corrosion is much more complicated in theory, which is associated with not only the carbon oxidation but also the dissolution of various nitrogen moieties.^20^ The commonly used in situ Fourier transformed infrared spectroscopy (FTIR) technique provides the potential-resolved information of the oxygen-containing functionalities on the carbon surface, yet neither directly reflecting the corrosion progress nor revealing the change of nitrogen moieties.^21^ Differential electrochemical mass spectroscopy (DEMS) gives CO_2_ signal during carbon corrosion, which is supposed to be an ideal approach of online monitoring.^14,22−24^ Yet, the formed acidic gases (e.g., CO_2_ and, if any, NO_2_) will be readily absorbed by an alkaline electrolyte; modifying DEMS cells, such as via coupling an online acidifying unit, is a promising solution, but they still suffer from the relatively low signal-to-noise ratio.^22^ Thus, despite the broad applications of NC in alkaline media, the dynamic progress of N dissolution and the associated carbon corrosion of NC remain unclear.

In this work, we reported the operando and ex situ examinations of denitrogenation and corrosion of NC under high anodic potentials. The combined spectroscopic and electrocatalytic studies demonstrated nearly complete dissolution of N atoms. Via coupling a gas-diffusion electrode with tailored triple-phase boundaries in a DEMS cell, we managed to detect various carbon and nitrogen oxides during NC corrosion. The oxidations of both carbon and nitrogen domains initiated essentially simultaneously (ca. 1.2 V vs RHE), both of which were 0.3 V lowered than that of water oxidation. Our density-functional theory (DFT) simulation suggested that the free energy change of N oxidation was close to that of C oxidation. We also showed that the NC corrosion can be suppressed by electrodepositing a metal hydroxide with optimized loading on the surface.

## 2 Results and Discussion

### 2.1 Electrochemical Response under High Anodic Potentials

**Figure 1:**
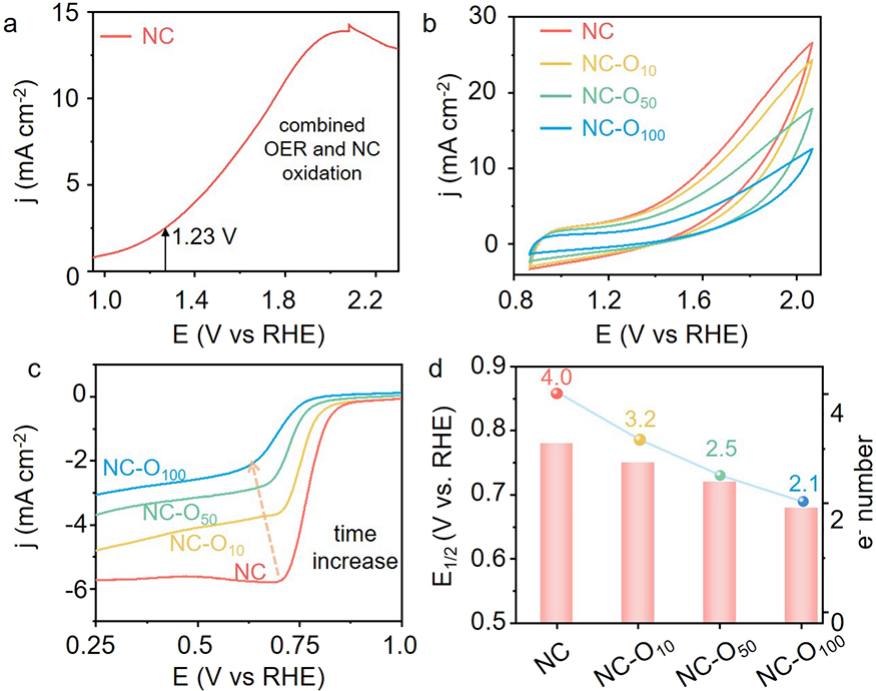
(a) LSV curve of NC with Faradaic currents from both OER and NC oxidation; (b) CV curves of NC after 0, 10, 50, and 100 cycles from 0.87 to 2.03 V vs RHE. (c) LSV curves of NC after various CV cycles at 1600 rpm; (d) comparison of *E*_1/2_ and electron transfer number of NC in ORR. All measurements are performed in oxygen saturated 0.1 M KOH electrolyte at 25 °C using a rotating disc electrode.

The NC material in this study was synthesized via the previously reported strategy using magnesium nitrilotriacetate as the precursor, which is suitable for a large-scale (kg level) production with excellent and stable ORR performance in both rotating disc electrode (RDE) and in anion-exchange membrane fuel cells.^25,26^ We first examined the electrochemical response of this NC under high anodic potentials in the oxygen-saturated 0.1 M KOH electrolyte. Figure 1a shows the linear-sweep voltammogram (LSV) from 0.9 to 2.3 V (vs RHE and hereafter) which simulates the scenarios during operations in the oxidizing environment.^6,27−29^ The apparent current increase can be ascribed to the oxygen evolution reaction (OER). Yet, there were two catches in the curve: (1) the anodic current density reached 2.15 mA cm^–2^ below 1.23 V, indicating the presence of side reactions; (2) the current started to increase abnormally when the voltage passed 2 V, implying the possible electrochemical passivation of the electrode material (the passivation plateau was prominent in the very first scan). This phenomenon has also been observed during the oxidation of other carbon materials, but it remains unclear how the passivation reaction proceeds dynamically.^30^

As a single LSV scan to the high anodic potential is not likely to induce a significant compositional and structural change of NC, we then carried out cyclic voltammetry (CV) in the potential window from 0.87 to 2.03 V. Figure 1b shows the CV voltammograms after 10 (NC-O_10_), 50 (NC-O_50_), and 100 cycles (NC-O_100_). The peak current density decreased progressively with the increase of the cycle number. Interestingly, in the LSV scan at 1600 rpm, a clear negative correlation between the cycle number and the ORR half-wave potential (*E*_1/2_) was observed (see Figure 1c). This agrees with the electrochemical impedance spectroscopy (EIS) data shown in Figure S1: the charge-transfer resistance increased sharply with the increase of cycle numbers. The onset potential (*E*_0_) together with the limiting current density also decreased when the cycle number rose. We thus performed the RDE test at different rotating speeds and obtained the Koutecky–Levich plot (see Figure S2). Figure 1d summarizes the key performance indicators as a function of cycle number. The half-wave potential shifted from 0.79 V for pristine NC to 0.56 V for NC-O_100_. The calculated numbers of electrons transferred (*n*) also decreased gradually from 4.0 to 2.1, implying the ORR mechanism has been altered completely from 4e^–^-transfer pathway to the generation of peroxide with 2e^–^ transfer which is similar to the catalytic behavior of pristine graphite without heteroatom doping.^8,31^

From the abovementioned electrochemical data, we can infer that the high anodic bias might indeed induce physicochemical properties change of nitrogen-doped carbon materials on the electrode. The substantial decrease in the ORR activity should be linked to the change of N moieties (e.g., surface blockage, N dissolution, and catalyst detachment from the electrode) which are believed to be the active sites.^11^ Hence, confirming and understanding the dynamic evolution of the active sites in response to the variation of potential bias requires both ex situ and operando characterizations.

### 2.2 Ex Situ Characterizations of the Degraded NC

**Figure 2:**
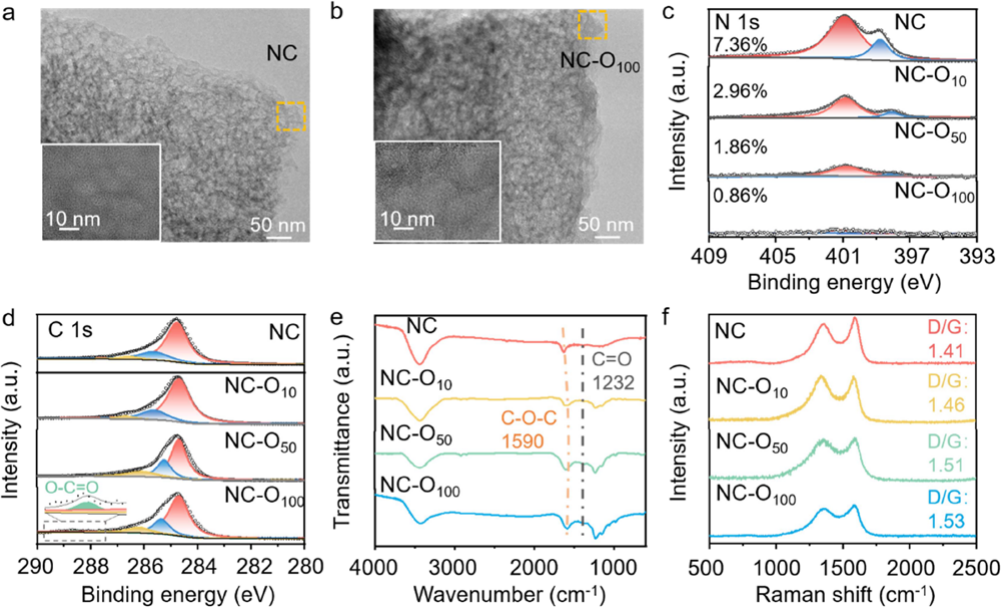
Comparison of NC, NC-O_10_, NC-O_50_, and NC-O_100_ properties containing (a, b) TEM micrographs of NC and NC-O_100_, the insets demonstrate the mesopores; (c, d) deconvoluted core-level XPS spectra of N 1s and C 1s; (e) FTIR spectra; and (f) Raman spectra.

The morphological and structural changes of NC, in comparison with NC-O_10_, NC-O_50_, and NC-O_100_, were first investigated using a transmission electron microscope (TEM, see Figures 2a,b and S3). No apparent changes were observed between the pristine NC and oxidized samples. In the high-resolution TEM (HRTEM) micrographs, the mesoporous structure of pristine NC was clearly visible in the inset of Figure 2a. Conversely, we noticed that such a structure clearly collapsed in NC-O_100_ as the mesopores were hardly observable. This observation of local structure was aligned with the N_2_ adsorption study. The pristine NC showed a typical H3 and H4 adsorption–desorption hysteresis loop, confirming the presence of abundant mesopores (see Figure S4). This loop disappeared in NC-O_100_, in agreement with the diminish of mesopores. The pore size distribution analysis in Figure S4b reveals that the electrochemical oxidation triggered the formation of more micropores. This electrochemical effect on the pore structure of carbon is similar to that caused by chemical oxidations as reported in the literature.^32,33^

We then used X-ray photoelectron spectroscopy (XPS) to examine the surface change. As shown in Figure 2c, the pristine NC showed a strong N signal with 7.36 at % of nitrogen moieties. The N 1s core-level spectrum can be deconvoluted as the peaks at 398.4 and 400.5 eV were assigned to pyridinic N and graphitic N, respectively.^31,34^ These two species were widely documented to be active for ORR. The nitrogen moieties were removed gradually as the oxidation cycles increased. In particular, the pyridinic N and graphitic N species seemed to disappear more rapidly from NC-O_10_ to NC-O_100_. Finally, NC-O_100_ showed a completely different spectrum: the total N content dropped to 0.86 at % and the pyridinic N moiety was almost undetectable (see the summary in Table S1). This strong contrast of N doping ratio change was also reflected by the elemental analysis using the energy-dispersive X-ray spectroscopy (EDX) shown in Figure S5. As EDX offers bulk properties, we inferred that the oxidation of N-moieties, instead of the blockage, on the surface occurred.

In addition to the denitrogenation reaction, the oxidation of carbon was also prominent. The C 1s spectrum of NC showed the presence of C–C bond at 284.3 eV, C–O–C bond at 285.6 eV, and C=O bond at 286.7 eV.^34,35^ The oxygen-containing functionalities became much more evident in NC-O_100_. In particular, a new peak at 288.6 eV was detected (see the inset in Figure 2d), which corresponded to the O–C=O bond.^35^ Carbon corrosion was also visible using the Fourier-transformed infrared spectroscopy (FTIR) shown in Figure 2e. The vibrational bands of NC centered at 1630 and 3500 cm^–1^ were due to the presence of water; no other functionalities were detected. As the oxidation cycles increased, new peaks at 1590, 1232, 1055, and 974 cm^–1^ emerged, which were connected to the formation of oxygen-containing functionalities such as C–O–C, C=O, and quinone groups.^36−38^ In the Raman spectrum, the ratio between ordered (G-band) and disordered graphite planes (D-band) changed after the electrochemical oxidation. The D/G ratio increased progressively from 1.41 for pristine NC to 1.53 for NC-O_100_ (see Figure 2f and the deconvoluted spectra in Figure S6), implying that the oxidation has created more defective domains in NC via transforming a portion of ordered graphite into the amorphous carbon.^39^ This observation agrees with the selective area electron diffraction (SAED) patterns shown in Figure S7 and the fact that more micropores were formed after the oxidation (cf. pore size distribution).

Based on the ex situ characterization results, we can conclude that the high anodic potentials applied on the NC indeed led to the materials corrosion, partially destructing the mesoporous structure and creating more micropores/amorphous domains. In addition to the surface reconstruction, leaching of both nitrogen and carbon atoms occurred on the surface of NC, yet the removal of N-moieties was more apparent. The ORR activity degradation of NC-O_*x*_ was pertinent to the removal of the N dopants. However, several fundamental questions remain: (1) when (at which potential) did the leaching occur? did the oxidation of N and C atoms happen at the same potential? (2) what are the leaching products? (3) what is the pathway/mechanism of denitrogenation?

### 2.3 Operando DEMS Analysis

**Figure 3:**
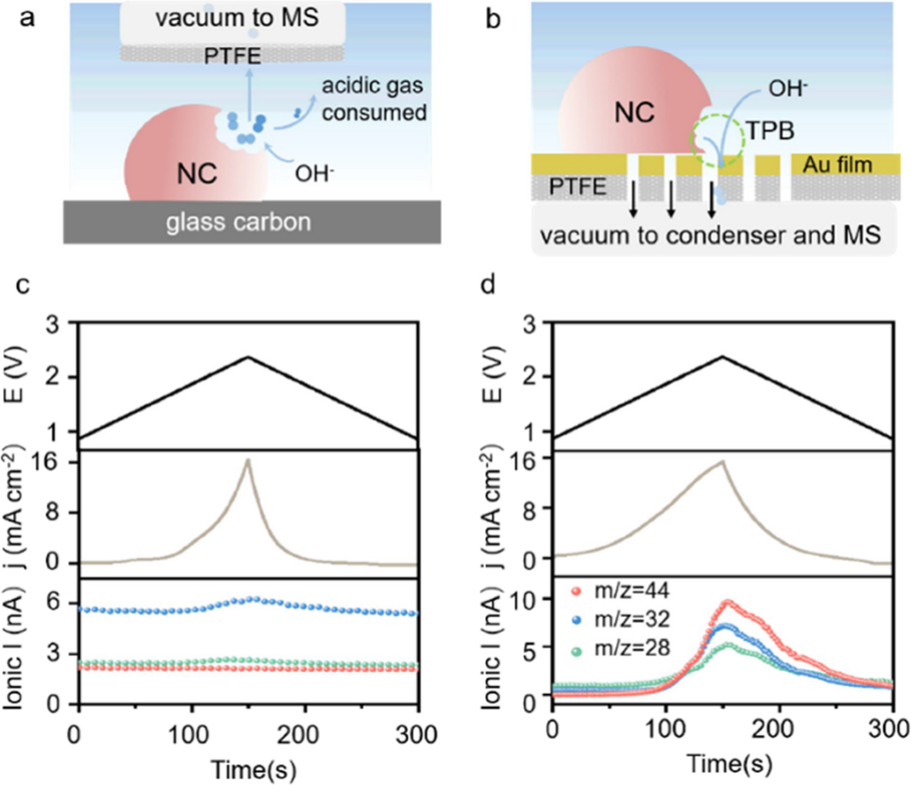
(a, b) Schematic configurational comparison of two DEMS cells; the CV curves and the corresponding ionic currents obtained from (c) probe cell and (d) Au film cell; measurements are performed in oxygen saturated 0.1 M KOH electrolyte at 25 °C with a scan rate of 10 mV s^–1^.

In an effort to tackle these questions, we used the differential electrochemical mass spectrometry (DEMS) to monitor the gaseous and volatile corrosion products in situ. Considering the reaction between the KOH electrolyte and the possibly evolved acidic gases (i.e., CO_2_ and NO_2_), we employed two distinct DEMS cells for this study (Figure 3a,b). One of the cell configurations contains a membrane-inlet probe with the tip covered by a Teflon (PTFE) membrane separating the electrolyte and the vacuum environment inside (see the schematic in Figure 3a). This probe was placed 50 μm on top of the glassy carbon electrode covered by the pristine NC catalyst. In the CV cycle between 0.87 and 2.34 V (vs RHE) as shown in Figure 3c, the evolution of O_2_ (*m*/*z* = 32) and CO (*m*/*z* = 28) could be detected by the mass spectrometer (MS), yet other signals (e.g., CO_2_ with *m*/*z* = 44) were hardly measured. The generated acidic gases would readily react with the surrounding KOH electrolyte during the diffusion to the probe’s tip and thus were undetectable by the MS.

The other cell configuration used a porous Au-coated polytetrafluoroethylene (PTFE) film as the working electrode on top of which the NC catalyst was deposited. On the opposite side, a frit was used to offer the mechanical support in the vacuum (see Figures 3b and S8). A detailed configuration of the system can be found in the Supplementary Information (SI). On this electrode, a distinct three-phase boundary (TPB) was created where the NC, pore, and KOH electrolyte met each other. The evolved gases at the TPBs would be continuously sucked into the vacuum tube via the pores without suffering prolonged interactions with the electrolyte. Theoretically, it offered opportunities for detecting all gases generated in the alkaline electrolyte; maximizing the length of the TPB would increase the sensitivity of the detection. We hence optimized the catalyst loading together with the porous properties of the electrode film, and strong MS signals of both CO_2_ and CO (see Figure 3d) in the CV cycle between 0.87 and 2.34 V (vs RHE) were then recorded in this DEMS cell.

**Figure 4:**
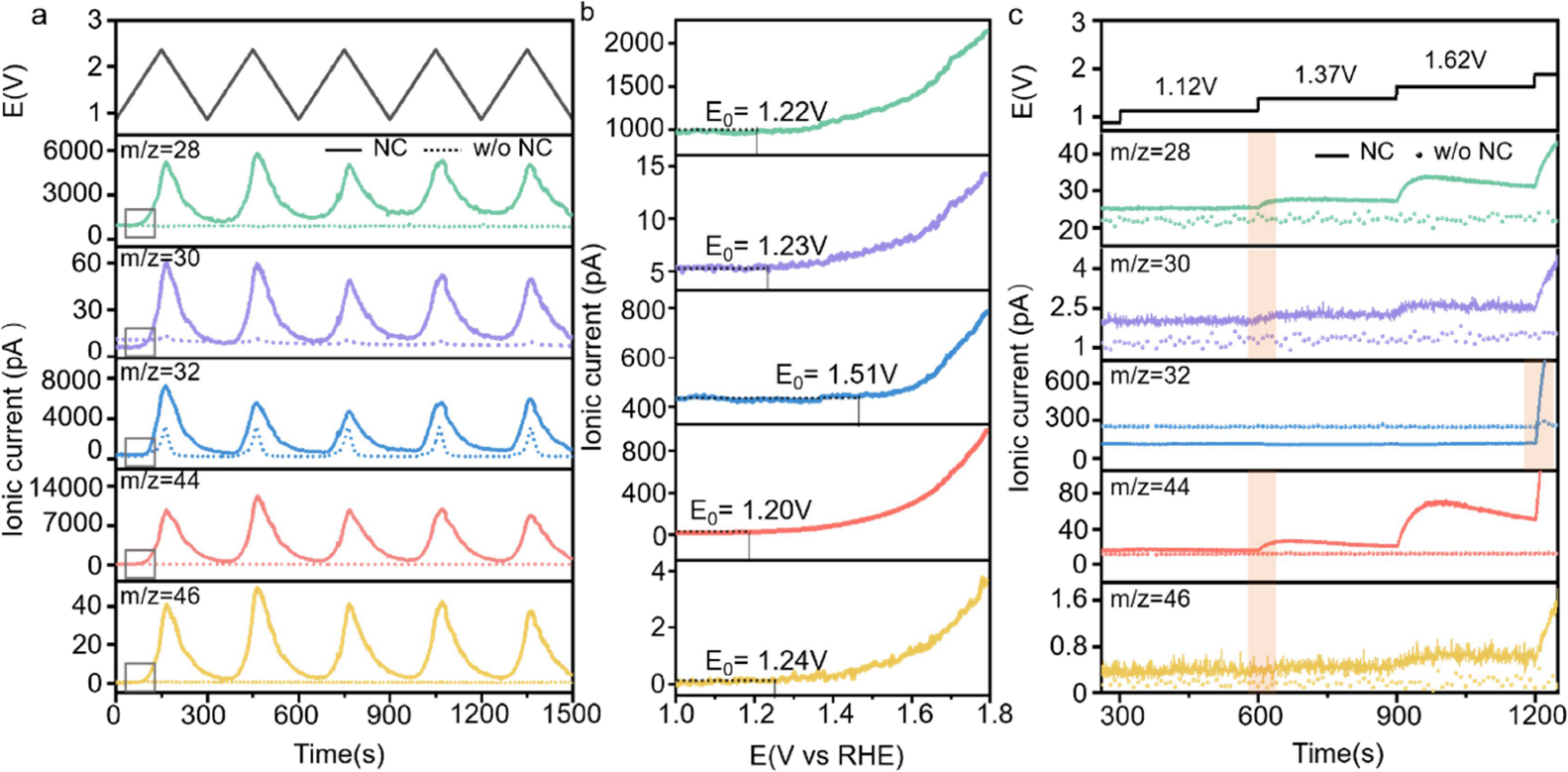
(a) Multiple CV cycles and the corresponding ionic currents obtained in the Au-film cell with (solid line) and without (dotted line) the deposition of NC; (b) enlarged graphs of the square area in (a) showing the onset potentials of each gas evolution reaction; (c) potentiostatic analysis with stepwise potential increase of 0.25 V per step and the corresponding ionic current. All measurements are performed in oxygen saturated 0.1 M KOH electrolyte at 25 °C, the voltage window is between 0.87 and 2.12 V vs RHE, and the scan rate is 10 mV s^–1^.

This observation encouraged us to further affirm the behaviors of NC under high potential biases by performing both potentiodynamic and potentiostatic analyses. In the multiple CV cycles, the working electrode without NC catalyst only showed MS signal at *m*/*z* = 32 (Nafion binder was still applied to exclude its influence over MS signals), implying the formation of O_2_ (see the dotted line in Figure 4a). Conversely, the NC electrode demonstrated 5 signals (*m*/*z* = 28, 30, 32, 44, 46), similar to the results in the acid electrolyte (see Figure S9). Their profiles perfectly matched the CV cycles. The strong signals at *m*/*z* = 28, 32, and 44 were ascribed to the formation of CO, O_2_, and CO_2_. The ionic currents from CO_2_ and CO were comparable with that from O_2_, implying that the corrosion of NC was indeed a major electrochemical reaction during CV cycles.

We also observed weak yet periodic signals at *m*/*z* = 30 and 46 which corresponded to the evolution of NO and NO_2_ gases, respectively. This result was aligned with that from the previous work in which ex situ techniques revealed that NO_3_^2–^ was one of the typical NC electro-oxidation products.^20^ To the best of our knowledge, this is the first experimental result reporting the formation of NO and NO_2_ during NC oxidation in alkaline conditions using operando techniques. Computational modeling also suggested that these oxidation reactions were thermodynamically favorable (vide infra). NO_*x*_ formation from other sources, such as the oxidations of dissolved N_2_, NH_3_, or NH_4_^+^, was ruled out based on the following reasons. On the one hand, the DEMS cell was purged with high-purity Ar prior to the examination, the Nernst potential for N_2_ oxidation was much higher than the observed onset potential of NO_*x*_ evolution; on the other hand, no ammonia fragment has been recorded by the MS (the most typical one is NH^+^ with *m*/*z* = 15). Thus, the residual ammonia in the electrolyte, if any, was unlikely to trigger the observed MS signal change. In fact, we intentionally added various concentrations of NH_4_^+^ into the electrolyte, no additional oxidation peak was observed in the CV cycles (see Figure S10). We therefore concluded that the electrochemical denitrogenation of NC in KOH resulted in the formation of NO and NO_2_.

The onset potentials (*E*_0_) of different gases varied as shown in Figure 4b. We defined *E*_0_ by finding the voltage point from the ionic current profile where the current started to rise above the baseline. The onset potentials for CO, CO_2_, NO, and NO_2_, were essentially identical (1.22 ± 0.02 V vs RHE), indicating that the electrochemical removal of C and N atoms from NC might be coupled (vide infra). O_2_ evolution started at a much later stage with *E*_0_ = 1.51 V vs RHE. The *E*_0_ of each product did not change much after 4 CV cycles (Figure S11). But the *E*_0_ of O_2_ decreased slightly, which might be due to the surface reconstruction of NC in such a way that facilitated the oxygen evolution reaction.^40^

The steady-state test results using potentiostatic analysis were consistent with the potentiodynamic study above (see Figure 4c). The ionic currents were monitored in response to the stepwise potential increase (from 0.87 to 2.12 V vs RHE, 250 mV interval, 300 s at each step, a full profile was shown in Figure S12). The dotted signals came from the control group without depositing NC. No MS signals were observed at 0.87 and 1.12 V for the NC electrode. Starting from 1.37 V, CO, CO_2_, NO, and NO_2_ signals appeared simultaneously. The NO_2_ signal was relatively much weaker compared with that obtained in the potentiostatic study at the identical potential bias, suggesting that this 4e^–^-transfer oxidation of N atom to NO_2_ might be kinetically more challenging compared with the oxidation to NO. A slight decrease of all the ionic currents was seen as a function of time at each voltage step, this could be attributable to the surface passivation of NC (e.g., formation of oxygen-containing functional groups, see XPS and FTIR results above). The formed passivation layer blocked the reactive domains, inhibiting the oxidation of materials underneath. Note that the stable oxygen evolution was seen till 1.87 V vs RHE. Conversely, oxygen’s ionic current did not decrease much, but increased (at high-voltage window) as a function of time at each voltage step. We deduced that the self-reconstruction of the surface occurred under potential bias, which generated more amorphous domains and oxygen-containing functionalities. This facilitated the OER yet suppressing the oxidation of NC and hindering the ORR catalysis. We repeated this stepwise test three times (see Figure S13). While the NC corrosion dominated in the first cycle, OER became increasingly prominent in the following cycles. The ionic currents for CO, CO_2_, NO, and NO_2_ all decreased sharply, further supporting the surface passivation theory at high voltages.

### 2.4 Understanding the Denitrogenation Pathway

**Figure 5:**
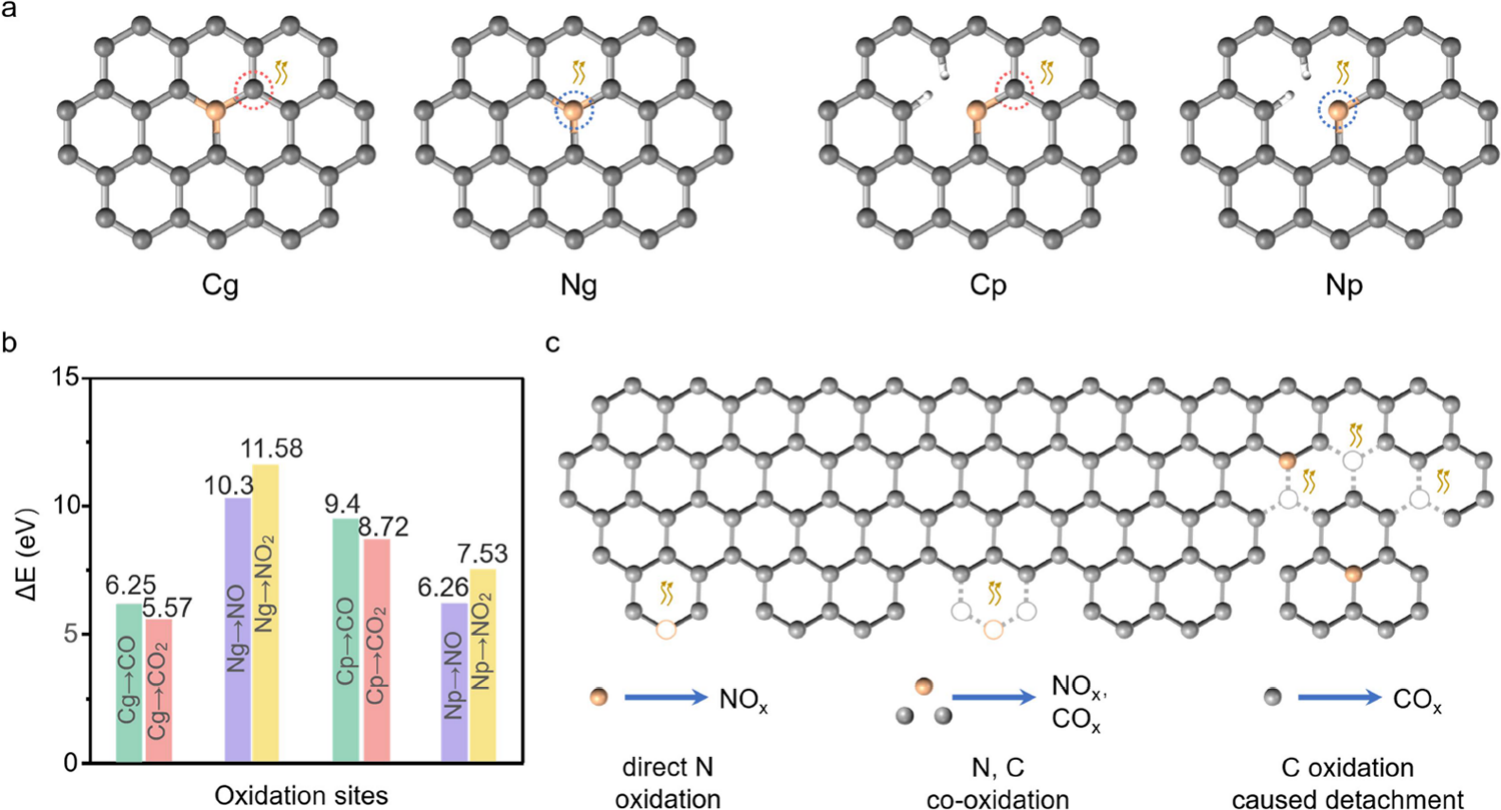
(a) Configurations of selected oxidation sites of NC used in the DFT calculation; N atoms are orange in color; (b) plot of the calculated oxidation free energy of various moieties; and (c) hypothetic routes of NC denitrogenation.

As the carbon oxidation pathway and mechanism have been investigated before,^41^ we are particularly interested in elucidating the pathway of denitrogenation. Density functional theory (DFT) calculation^42^ was applied to examine the free energy barrier (Δ*E*) of the possible oxidation reactions in NC. We defined four distinct reaction sites with either N or C as the reaction atom: pyridinic N (Np), graphitic N (Ng), the nearest carbon atom of Np (Cp), and the nearest carbon atom of Ng (Cg). The schematic structures are depicted in Figure 5a with the detailed unit cell shown in Figure S14. A total of 8 reactions, generating either CO_*x*_ or NO_*x*_ (*x* = 1, 2) were thus considered
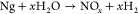
1
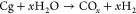
2
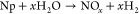
3
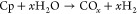
4

The free energy barrier (Δ*E*) plot was shown in Figure 5b. In the graphitic structure, the oxidation of C atoms was much easier than that of N atoms. The Δ*E*(Cg) of the reactions to CO and CO_2_ were 6.25 and 5.57 eV, respectively, while the Δ*E*(Ng) of the reactions to NO_*x*_ exceeded 10 eV. In the pyridinic structure, however, direct oxidation of N atoms was favored, the Δ*E*(Np) of the reactions to NO and NO_2_ were 6.26 and 7.53 eV, respectively. The Δ*E*(Cp) of the reactions to CO_*x*_ exceeded 8.7 eV. Note that in both structures, the carbon oxidation to CO_2_, rather than to CO, was energetically preferred. Yet, the nitrogen oxidation to NO_2_, rather than to NO, was energetically more difficult. This result was in line with the prediction using Nernst potentials.

Based on the DFT results together with the above ex situ and operando characterizations, we proposed three basic routes of NC denitrogenation as illustrated in Figure 5c. The first one was the direct oxidation of pyridinic nitrogen atoms at the edge. Previous work demonstrated that OH^–^, together with the sequentially formed other oxidative intermediates at high potential, would be preferentially adsorbed at Cp sites.^11,43^ The adjacent Np moieties were thus easy to be oxidized. As these moieties were believed to be the active sites of ORR, their disappearance would drastically undermine the ORR catalysis (see Figure 1). The second route involved the co-oxidation of C and N atoms. The corrosion initiated by the carbon atoms, creating more micropores while converting the adjacent N_g_ into N_p_ moieties in NC. Subsequently, the oxidation of these nitrogen atoms became energetically favorable and denitrogenation occurred. The last route was contributed solely by the oxidation of C atom which caused the structure disintegration of NC (e.g., the collapse of mesopores discussed above). Thus, NC fragments containing N moieties would detach from the bulk electrode, resulting in the denitrogenation of NC.

### 2.5 Alleviating NC Denitrogenation

From the mechanistic point of view, if the oxidation reaction sites could be mitigated away from the surface of NC, the corrosion might be alleviated. One of the most straightforward, and also commonly applied in the literature, approaches of optimizing NC is to form a hybrid structure with a second phase such as oxides, sulfides, or phosphides.^22,44^ Indeed, this strategy has been proven with excellent efficiency in terms of boosting the catalytic activity of NC in reactions such as OER and ORR.^45,46^ But the remaining question is: can we really suppress the denitrogenation and corrosion of NC under high anodic potentials in this structure? This intuitive question comes from two seemingly contradictory facts: on one hand, transferring the oxidation reaction sites from NC to the secondary phase would naturally suppress the corrosion of NC; on the other hand, the secondary active phase, e.g., Pt, might catalyze the oxidation of the carbon support at the interface.

**Figure 6:**
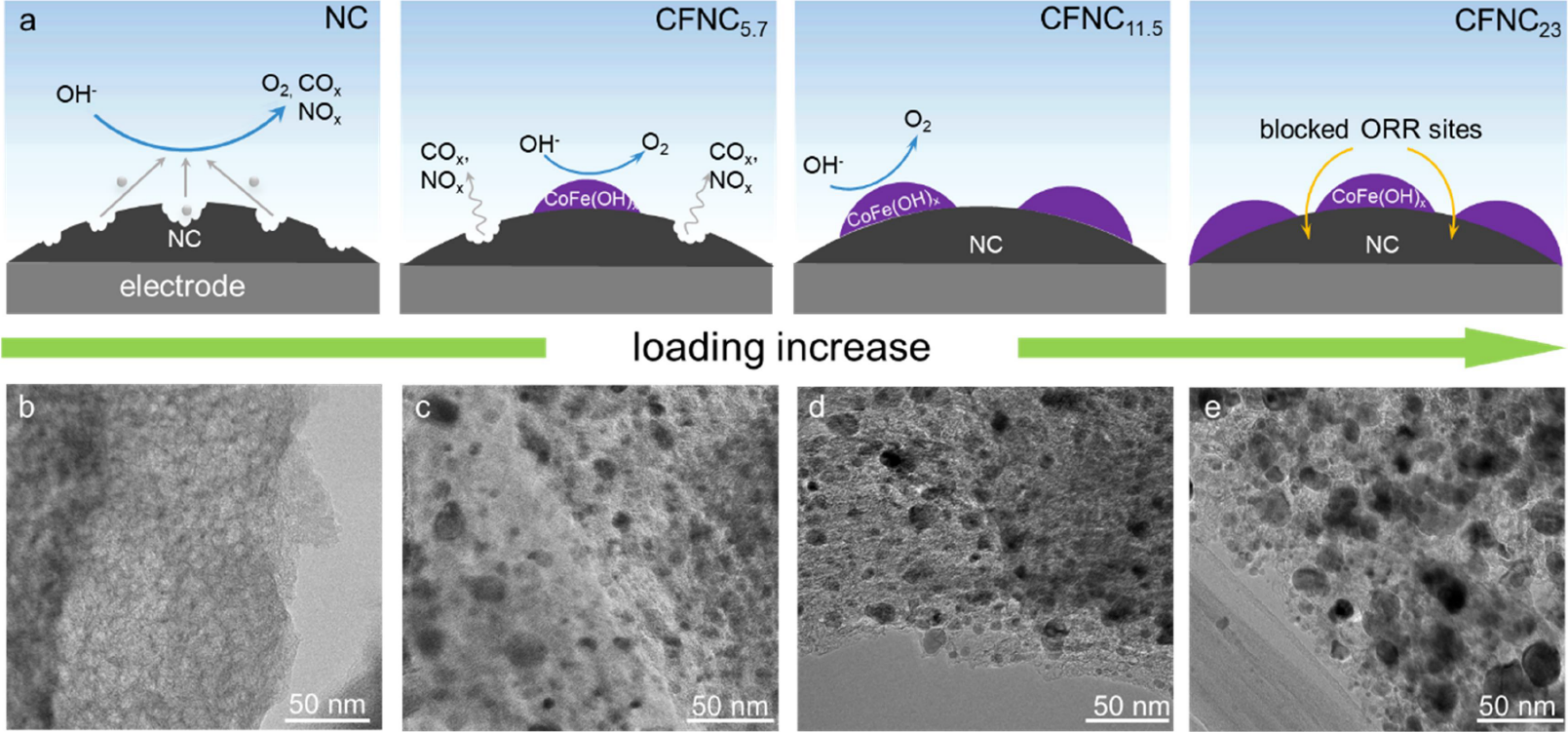
(a) Schematic illustration of CFNC with various loadings of CoFe(OH)_*x*_; and (b–e) corresponding TEM micrographs.

To investigate the possible roles of a secondary active phase on top of the NC, we constructed an NC-supported metal hydroxide (MOH, M = Ni, Fe, Co, and the corresponding binary ones) catalysts. Such hydroxides are well-known OER catalysts with excellent performance in adsorbing reaction intermediates and directing the formation of O_2_.^47,48^ To ensure their distribution on the “electroactive” regions, we used electrodeposition approach to apply MOH with various loadings (see the schematics in Figure 6a using supported CoFe(OH)_*x*_ nanoparticles, denoted as CFCN_*z*_, as the example; *z* represents the mass loading of CoFe(OH)_*x*_). In the corresponding TEM images in Figure 6b–e, we noted different coverages of CFCN nanoparticles on the surface. The EDX elemental mappings in Figure S15 reveal the uniform distribution of Fe and Co. The *d*-spacings from the high-resolution TEM micrograph are 1.59 and 1.72 Å, corresponding to the (110) plane of Co(OH)_2_ and the (220) plane of FeOOH, respectively. This agreed with the hydroxide XRD patterns shown in Figure S16.

**Figure 7:**
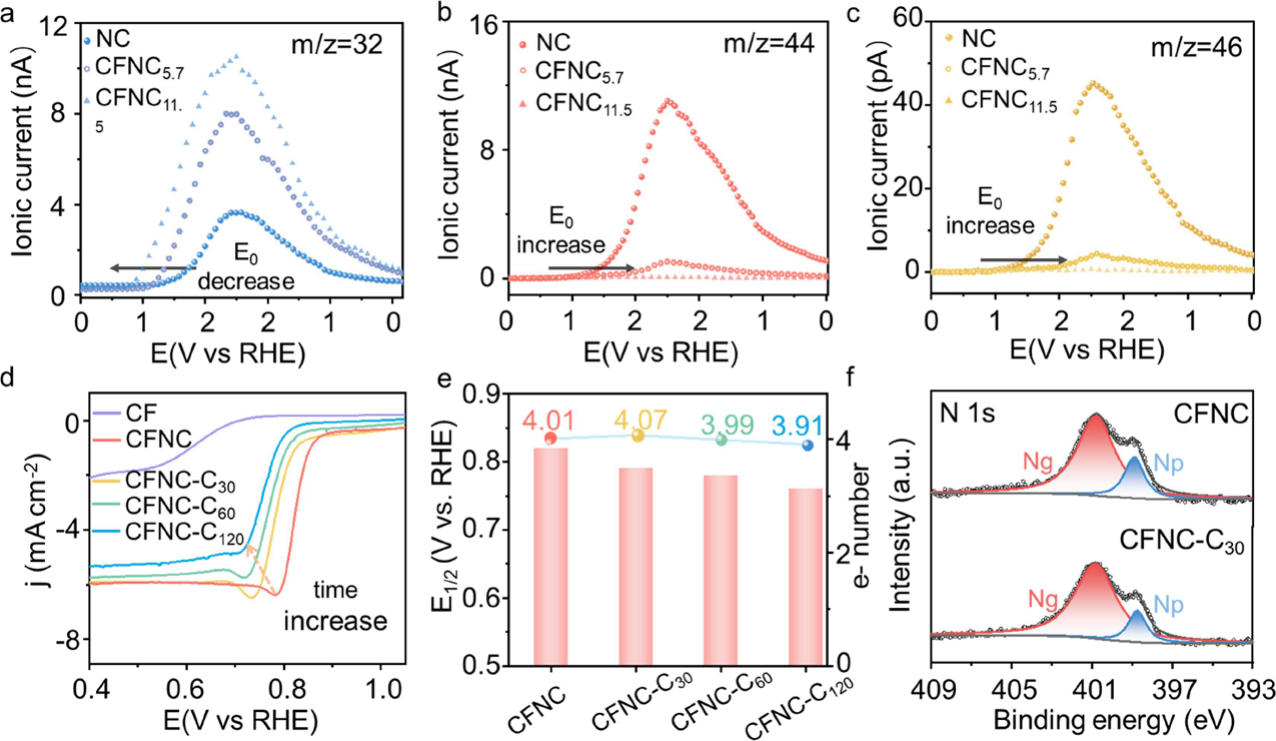
(a–c) Selected ionic currents from DEMS measurement of NC and CFNC with different loadings of metal hydroxides; the subscript in the sample name is the mass loading in percentage; (d) LSV curves of pristine and oxidized catalysts at 1600 rpm; the oxidation was performed via applying a constant potential at 2 V vs RHE for different periods of time, and the subscript in the sample name is time in minute; (e) comparison of *E*_1/2_ and electron transfer number; (f) N 1s core-level XPS spectra of CFNC and CFNC-C_120_. All measurements are performed in oxygen saturated 0.1 M KOH electrolyte at 25 °C.

We then again examined this hybrid electrocatalyst under high anodic potential using DEMS by applying CV cycles between 0.87 and 2.12 V. Figures 7 and S17 compare the MS ionic currents from the pristine NC, CFNC_5.7_, and CFNC_11.5_. Note that an identical amount of NC was deposited on the working electrode among different groups for better comparison. Apparently, the evolution of both CO_*x*_ and NO_*x*_ on the hydroxide-decorated NC was suppressed while OER became dominant. This effect became greatly prominent when the loading of CoFe(OH)_*x*_ reached 11.5% with nearly no DEMS signals of CO_*x*_ and NO_*x*_. However, higher loading (CFNC_23_) would impair the overall ORR performance as the ORR-inactive MOH blocked the active sites on NC (Figure S18 and the schematic in Figure 6a).

Another notable phenomenon was the onset potential of NC corrosion increased with the presence of hydroxides. For instance, *E*_0_(CO_2_) was ∼1.2 V for pristine NC while it became ∼1.9 V for CFNC_11.5_. However, completely inhibiting NC corrosion seems unlikely as the MS signals of NO and CO were still detectable at high voltages. This seemed inevitable as abundant nitrogen moieties remained exposed for catalysis. Figure 6a shows the hypothetic mechanism of suppressing NC corrosion in CFNC. At high anodic potentials, the possible oxidation reactions, together with the readily formed intermediates such as the highly oxidative radicals,^16^ were transferred from the NC surface to MOH surface. Such radicals were often short-lived, neither the indirect oxidation of the adjacent NC by the coming radicals via diffusion from MOH nor the direct oxidation of NC by having the reaction site on the NC surface can occur easily. Yet, at the interface between NC and MOH, corrosion of NC remained possible.

We lastly tested the ORR and OER performance CFNC_11.5_ together with NC decorated with other MOHs (see Figure S19 and Table S2). Pure CFNC was indeed a good OER catalyst yet demonstrating poor activity in ORR from the LSV curves. CoFe(OH)_*x*_ decorated NC outperformed the rest in the examined series. We thus used CFNC_11.5_ as the benchmark material to investigate the stability after oxidizing at 2 V vs RHE for different periods of time (CFNC_11.5_-C*y*, *y* indicates the time in min, see the *i*–*t* curve in Figure S20). In the LSV test using the RDE at 1600 rpm, the original performance of CFNC_11.5_ and NC was essentially identical, implying that the deposition of nanoscale hydroxides did not impair the ORR activity despite that the hydroxide itself was not active at all in ORR. The potentiostatic oxidation indeed jeopardized the ORR activity by decreasing the half-wave potential. However, this decrease was less than 10% while the number of electrons transferred maintained at ∼4 (see Figure 7).

XPS was carried out again to examine the nitrogen moieties in CFNC_11.5_ before and after oxidation. Both pyridinic and graphitic nitrogen moieties were sustained and their content were roughly identical to those in the fresh samples (see Figure 7f). Cobalt and iron were oxidized as oxyhydroxides started to appear in the spent catalyst (see Figure S21), implying that the MOH surface indeed became the oxidation sites. Together with the DEMS results, we concluded that the hydroxide phase on the surface of NC could suppress the denitrogenation and corrosion of NC at high anodic potential. While good ORR activity was sustained, a small degradation was still observed. Further research on optimizing the deposited secondary phase might be a feasible way of minimizing the leaching of N moieties.

## 3 Conclusions

In summary, we demonstrated that denitrogenation and corrosion of NC occurred at high anodic potentials in alkaline conditions. While destructing the mesopores and generating more micropores, this corrosion generated both CO_*x*_ and NO_*x*_ products simultaneously at ca. 1.2 V vs RHE which was >0.3 V below the water oxidation potentials. DFT calculations together with experimental results identified the possible corrosion sites and three possible basic routes of NC denitrogenation. Finally, we demonstrated that transferring the oxidation reaction sites to the deposited metal hydroxide was effective in suppressing the N leaching. This work demonstrates the dynamic evolution of NC under potential bias and might cast light on understanding and mitigating NC deactivation.

## Supporting Information Available

Additional TEM, XRD, Raman spectroscopy, N_2_-adosrption, XPS characterizations, and detailed DFT calculations together with more electrochemical test results (PDF)
