# Supplementary material for: Operando Studies of Electrochemical Denitrogenation and Its Mitigation of N-Doped Carbon Catalysts in Alkaline Media
Source: ACS Catal. 2023 Feb 9;13(5):2813–21. doi: 10.1021/acscatal.2c05590 (PMC9990068; doi:10.1021/acscatal.2c05590)
Supplement: Supplementary file 1 — cs2c05590_si_001.pdf [file cs2c05590_si_001.pdf]

Supplementary Information

Operando studies of electrochemical denitrogenation and its mitigation of N-doped carbon catalyst in alkaline media

Kai Zhao, [a] Shihao Han, [a] Le Ke,[a] Xiaoyu Wu,[a] Xiaoyu Yan,[a] Xiaojuan Cao,[a] Lingjiao Li, [a] Xiaoyi Jiang, [a] Zhiping Wang,[a] Huijun Liu,*[a] and Ning Yan*[a,b]

[a] School of Physics and Technology, Wuhan University, Wuhan, China.

[b] Van't Hoff Institute for Molecular Sciences, University of Amsterdam, Amsterdam, The Netherlands.

Corresponding Authors

*E-mail: Prof. N. Yan ning.yan@whu.edu.cn

Prof. H. Liu phlhj@whu.edu.cn

This file contains:

1. Experimental section

2. Supporting figures and tables

Experimental section:

1. Material synthesis. All chemicals were of analytical grade purity which were used without further purification.

Synthesis of N-doped carbon (NC). The NC material was synthesized via the previously reported strategy. Briefly, nitrilotriacetic acid (11.46g) and magnesium carbonate($(MgCO_3)_4Mg(OH)_2$, 5.8 g) were dissolved in water (150 mL) at 85°C. The solution was then cooled in an ice bath, in which 500mL ethanol was added slowing under vigorous stirring. The resulting suspension was kept in the ice both for 24 h to increase the yield of the magnesium nitrilotriacetate (MgNTA) precipitate. Then, the white precipitate was separated from the suspension and dried for 36 h in vacuum at 60 °C. The dried sample was pyrolyzed at 900 °C in argon (99.999% Ar) for 6 h. The obtained black powder was washed in citric acid solution several times to remove the MgO which was the pore-former. The remaining carbon precipitates were retrieved by filtration, washed by deionized water several times and dried for 5 h at 80 °C. Finally, a thermal treatment of the carbon was applied at 1000 °C in argon to increase the graphitization and decompose any adsorbed residual organics of the NC.

Synthesis of N-doped carbon (NC) with hydroxide cocatalyst. The hydroxide cocatalyst was synthesized via *in-situ* electrodeposition on glass carbon (GC) electrode coated with NC. Using $CoFe(OH)_x$ preparation as an example, stoichiometric amount of $Co(NO_3)_2 \cdot 6H_2O$and $Fe(NO_3)_2 \cdot 9H_2O$ (Co/Fe=2:1) were dissolved in deionized (DI) water and the total concentration of metal ions was 1 M. The obtained solution was further stirred for 30 min. The electrodeposition current density was 0.1 mA cm^{-2} and the mass loading of cocatalyst was controlled by the deposition time. After electrodeposition, the hybrid catalysts electrodes were rinsed with DI water several times before use.

2. Characterizations. X-ray diffraction (XRD) was carried out by the Rigaku Smartlab X-ray diffractometer using Cu Kα radiation at 40 kV and 44 mA. Transmission electron microscope (TEM) analysis was performed using a JEM-F200 TME coupled with a

high-angle annular dark filed (HAADF) detector and an energy dispersive x-ray spectroscopy detector. Raman spectroscopy was acquired by a Renishaw RM3000 Micro Raman system. The X-ray photoelectron spectroscopy (XPS) was carried out on a Thermo Fisher ESCALAB 250Xi instrument. The Fourier transform infrared spectroscopy (FTIR) was performed on a Bruker Invenio-R spectrometer. The nitrogen adsorption-desorption isotherms were obtained at 77 K using the JW-BK122W instrument from JWGB Sci. & Tech.

Electrochemical measurements. Electrochemical measurements were performed using a CHI 760E electrochemical analyzer (CHInstrument Inc.) and a rotating disc electrode setup from Pine Research Instrument. Inks of the carbon powders (1 mL ethanol,10μL Nafion® 5 wt% dispersion, 3.0 mg NC) were sonicated and dropcasted on a polished and cleaned glassy carbon electrode (Pine, Φ=5 mm). Hg/HgO electrode acted as the reference electrode and a Pt wire was used as the counter electrode. Prior to the electrochemical test, the electrolyte was purged with pure O_2 for 60 min to yield the O_2 saturated 0.1 M KOH solution. Post iR-compensation was applied at 95% value of the solution resistance which was obtained before each measurement. All reported current densities represented faradaic currents, with subtracted capacitive charging background currents. Electrochemical impedance spectroscopy (EIS) was measured potentiostatically with 5 mV perturbation in the frequency range of 0.04-10^5 Hz at the half-wave potential of oxygen reduction reaction, while bubbling oxygen and rotating at 1600 rpm. The number of moles of electrons transferred per mol O_2 (n) was estimated using the Koutecky–Levich equation below:

$$\frac{1}{J} = \frac{1}{B\omega^{1/2}} + \frac{1}{J_K}$$

where J is the measured disk current density (mA cm^{-2}), J_K is the kinetic current density; ω is the electrode rotation rate (rpm). The term B is given by

$$B = 0.2nFC_0D_0^{2/3}\nu^{-1/6}$$

Where n is the number of electron transfer; F is the Faradaic constant; C_0 is the bulk

concentration of O_2; D_0 is the diffusion coefficient of O_2; ν is the kinematic viscosity of the electrolyte.

Differential electrochemical mass spectrometry (DEMS) measurement. The operando differential electrochemical mass spectrometry (DEMS) system (Shanghai LingLu Instrument Corp., Ltd., China) contained a PrismaPlus quadrupole mass spectrometer from Pfeiffer Vacuum and custom-build Swagelok cells. A turbo vacuum pump (HiPace® 80, Pfeiffer) and condenser to remove the water vapor were coupled to the system. Note that the alkaline vapor must be removed completely to avoid the contamination and damage to the system. In the "probe-cell" configuration, a flow cell design was applied with continuous flow of 0.1 M KOH electrolyte to enable better mass transfer to the probe. In the "Au-film" cell, the gold coated porous PTFE film worked as the working electrode (0.4 cm^2) with dropcasted catalyst (see the ink composition above). The final film was ~27 μm thick with pores sizing ~25 nm by average. The NC loading was fixed at 0.625 mg cm^{-2}. To exclude the interference from the additives in the ink, the clean working electrode of the control experiment was also dropcasted with the ink without the addition NC catalyst. Ar was bubbled into the electrolyte prior to the experiment.

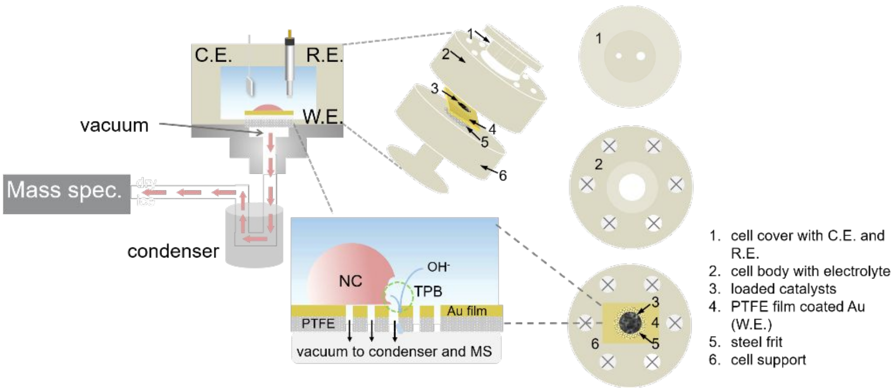

Schematic image of the DEMS (Au film cell) configuration.

3. Computational method. The first-principles calculation was performed using the density functional theory (DFT) as implemented in the Vienna ab-initio simulation

package (VASP). The exchange-correlation functional was in the form of Perdew-Burke-Ernzerhof (PBE) under the generalized gradient approximation (GGA). Electron exchange-correlation interactions were described using the generalized gradient approximation (GGA) method with PBE functional. The plane-wave cutoff energy was set to be 700 eV and the energy convergence criteria was 10^{-6} eV. We built a modified graphene supercell to describe the nitrogen doped carbon materials. Monkhorst-Pack k-mesh was used for sampling the Brillouin zone. We adopted a vacuum thickness of 30 Å to minimize the interaction between the monolayers and their periodic images.

The oxidation of N, C moieties followed the reactions below:

$$Ng/p + xH_2O \rightarrow NO_x + xH_2 \qquad \text{(eq. 1)}$$

$$Cg/p + xH_2O \rightarrow CO_x + xH_2 \qquad \text{(eq. 2)}$$

ΔE was defined as the free energies difference between the initial and final states; the structures of Ng, Np, Cg and Cp was illustrated in the main text. The calculated equation can be written as:

$$\Delta E = E_{products} - E_{reactants} \quad \text{(eq. 4)}$$

All the free energy of gas molecule was corrected follow the equation below:

$$E_{\mathrm{gas}}(T) = E_{slab} + \mathrm{ZPE} + H(T) + TS(T) \quad \text{(eq. 5)}$$

here the E_{slab} represents the calculated energy of the model, the ZPE represent the zero-point energy, the H(T) and S(T) are the enthalpy and entropy correction at the specified temperature T.

Supporting tables and figures

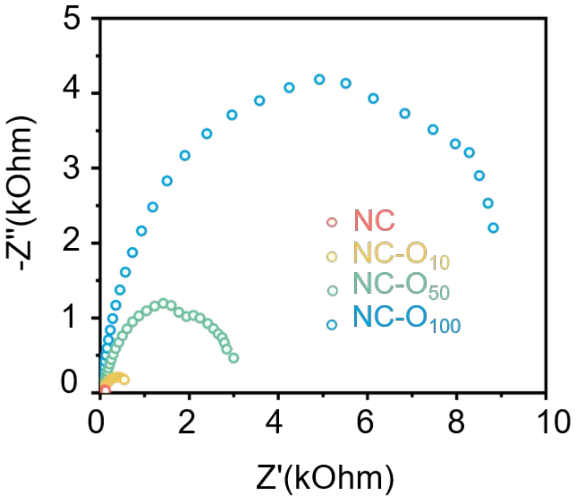

Figure S1. The EIS of NC and that of NC after various CV cycles obtained at the high-wave potentials of ORR.

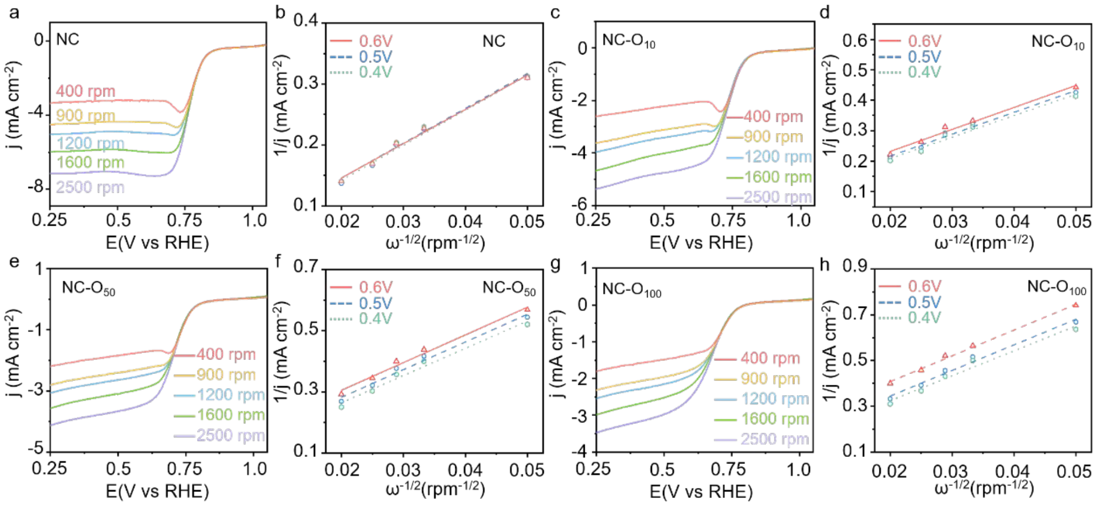

Figure S2. LSVs at various rotating speeds (400, 900, 1200, 1600, 2500 rpm) and the corresponding K-L plots of different NC samples.

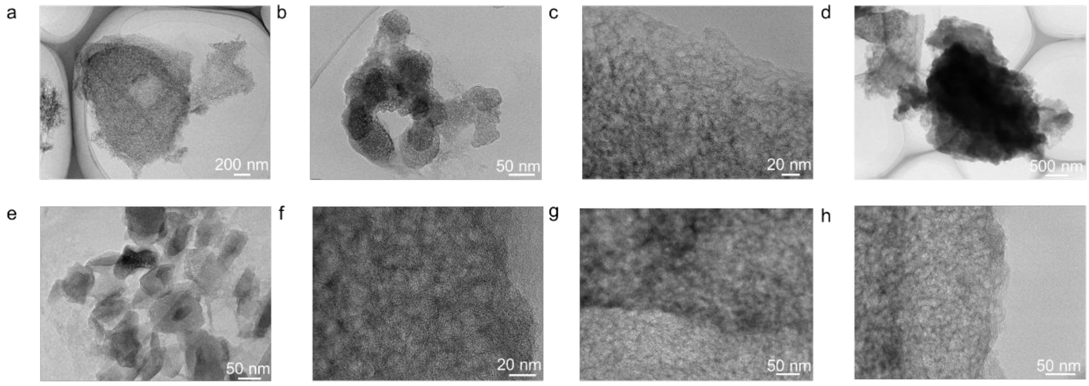

Figure S3. Morphology characterizations of (a-c) NC and (d-f) NC-O_{100} with different magnifications. (g) and (h) are the TEM images of NC-O_{10} and NC-$O_{50.}$

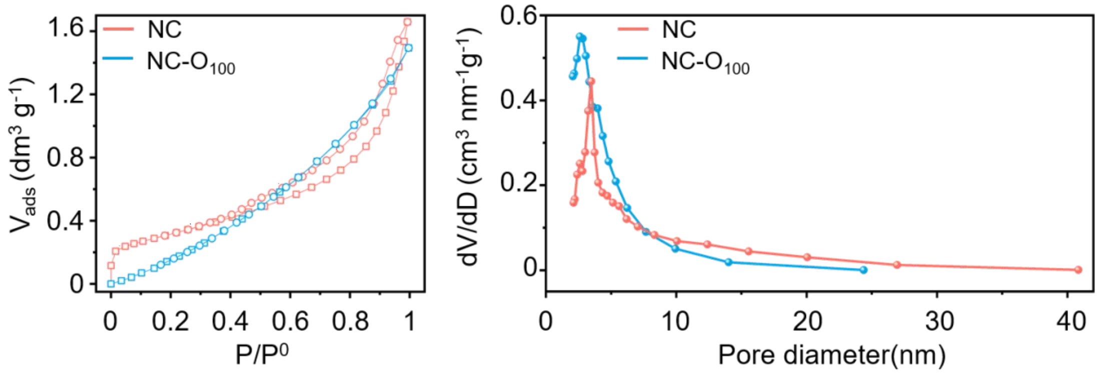

Figure S4. (a) Nitrogen-adsorption isotherm and (b)the pore size distribution of NC and NC-O_{100}.

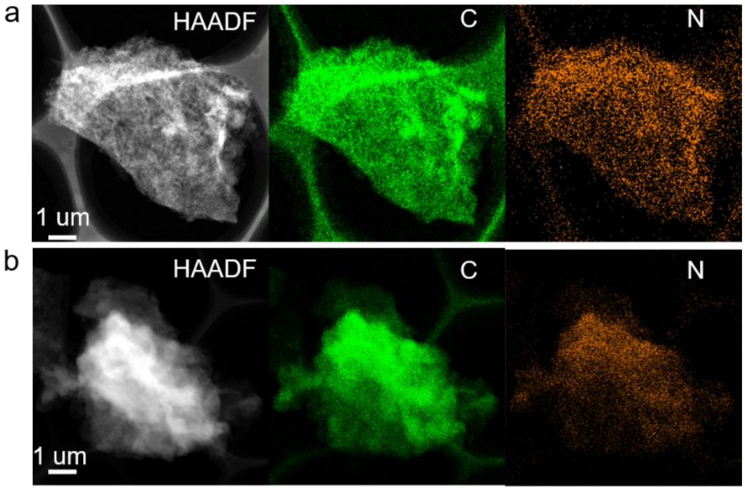

Figure S5. High-angle annular dark field (HAADF) images and EDX elemental mappings (C, N) of (a) NC and (b) NC-O_{100}.

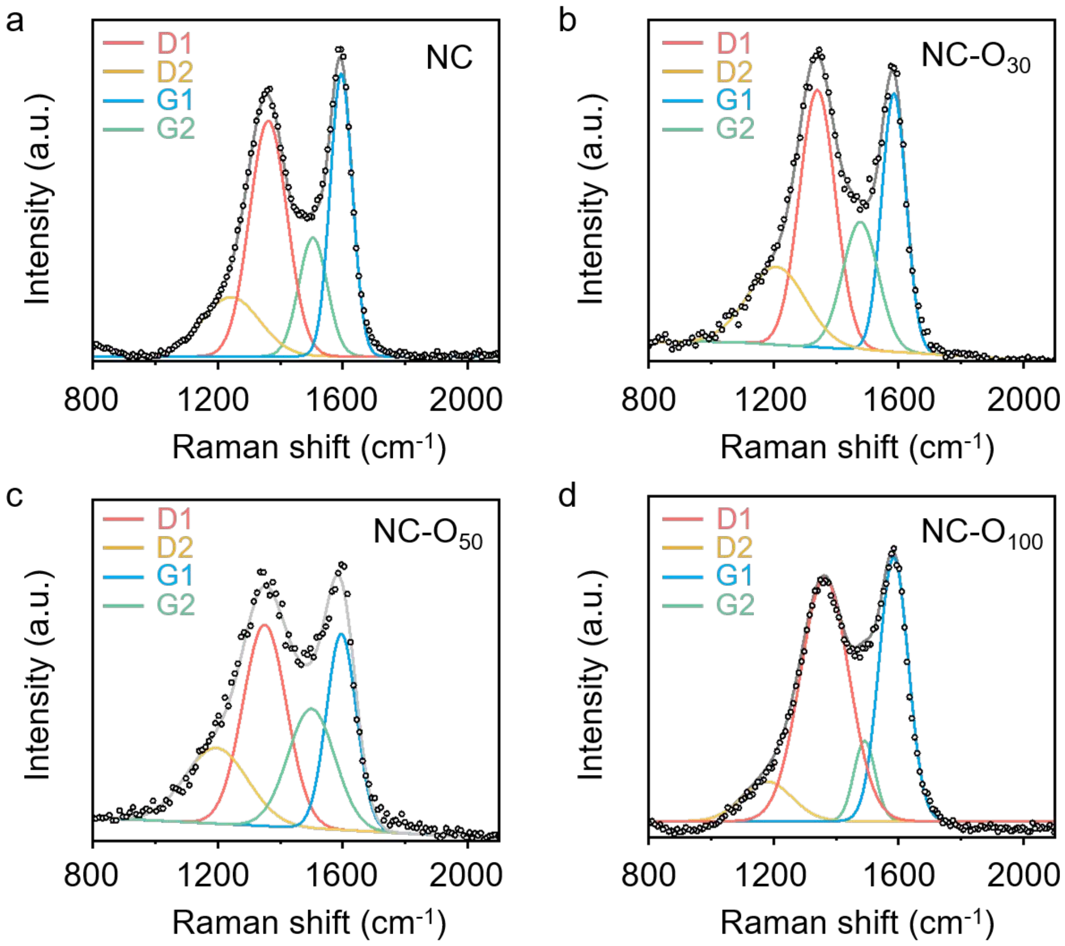

Figure S6. Deconvoluted Raman spectra of (a) NC and various oxidation samples: (b) NC-O_{10} , (c)NC-O_{50} , (d)NC-O_{100} .

All the Raman spectra are deconvoluted into four peaks named: D_1, D_2, G_1, G_2. The D_1 and G_1 peaks located at 1357 and 1570 cm^{-1} are indexed as the D and G bands of sp^2 carbon, representing the disorder carbon and the graphitic phase, respectively. The D_2 (nearly 1200 cm^{-1}) are usually assigned to disordered graphitic lattices (A_{1g} symmetry), polyenes or ionic impurities. The G2 always index to the amorphous carbon.

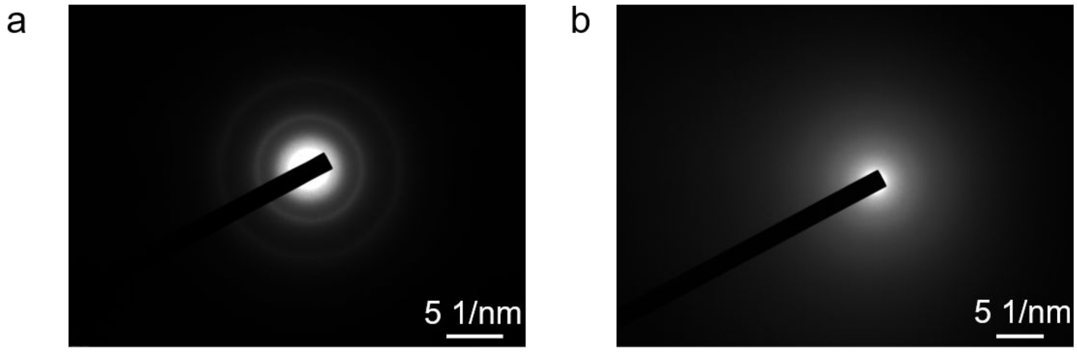

Figure S7. Selected area electron diffraction (SAED) patterns (scale bar: 5 nm^{-1}) of NC and NC-O_{100}

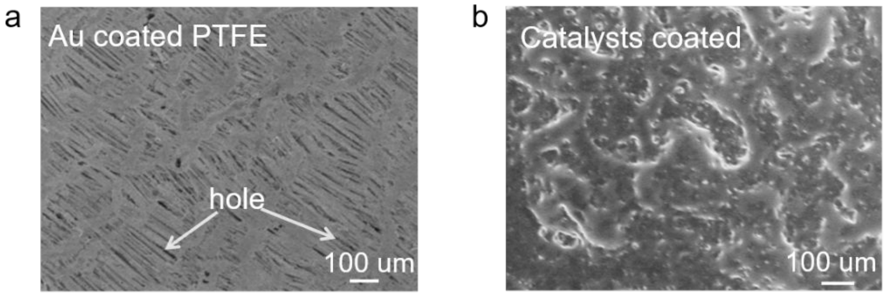

Figure S8. The SEM image of Au coated PTFE film and the prepared working electrode with catalysts.

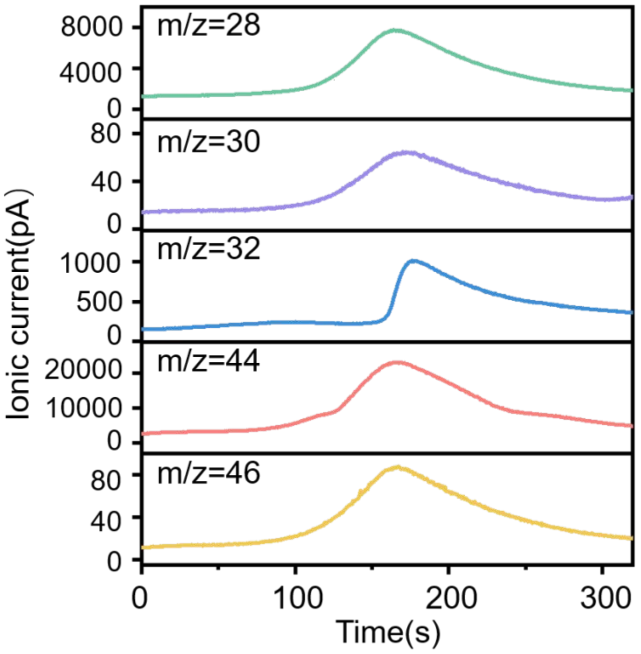

Figure S9. The MS signals of NC in acidic electrolyte (0.1M $HClO_4$).

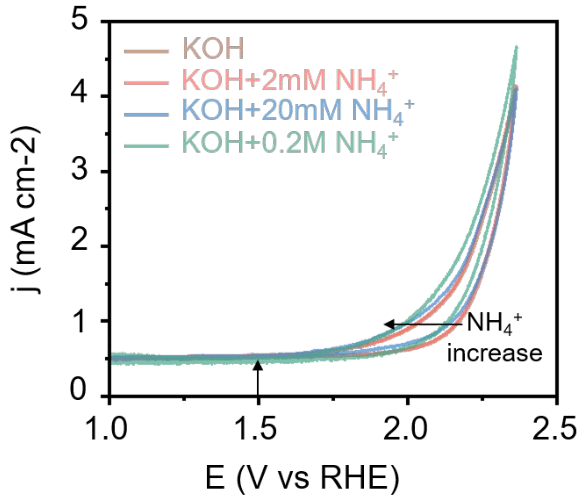

Figure S10. The CVs of NC electrode in 0.1M KOH containing various concentrations of NH_4^+.

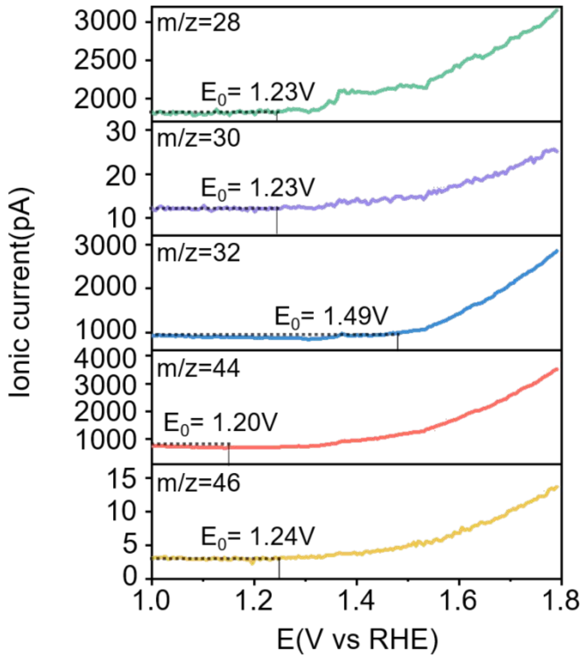

Figure S11. The amplified MS signals in the fourth CV cycle.

After 4 cycles, the onset potential of each product did not show obviously change. The E_0 of oxygen evolution (m/z=32) was slightly reduced.

.

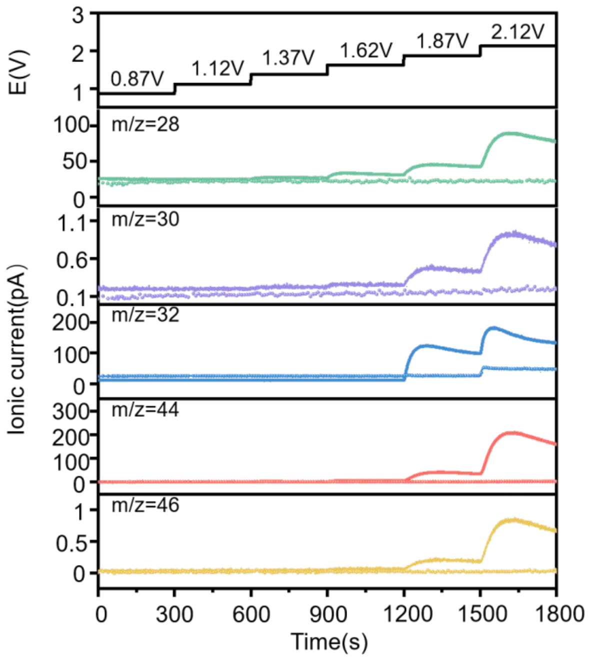

Figure S12. Potentiostatic analysis with stepwise potential increase of 0.25V per step and the corresponding ionic currents. Results were obtained in the Au-film cell with (solid line) and without (dotted line) the deposition of NC.

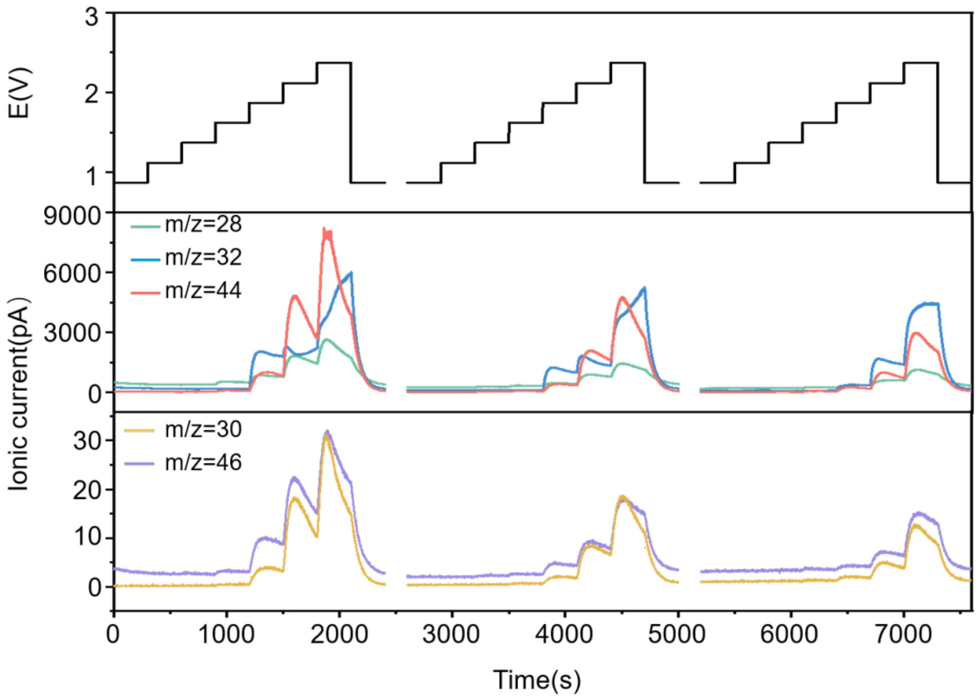

Figure S13. Repeated potentiostatic analysis with stepwise potential.

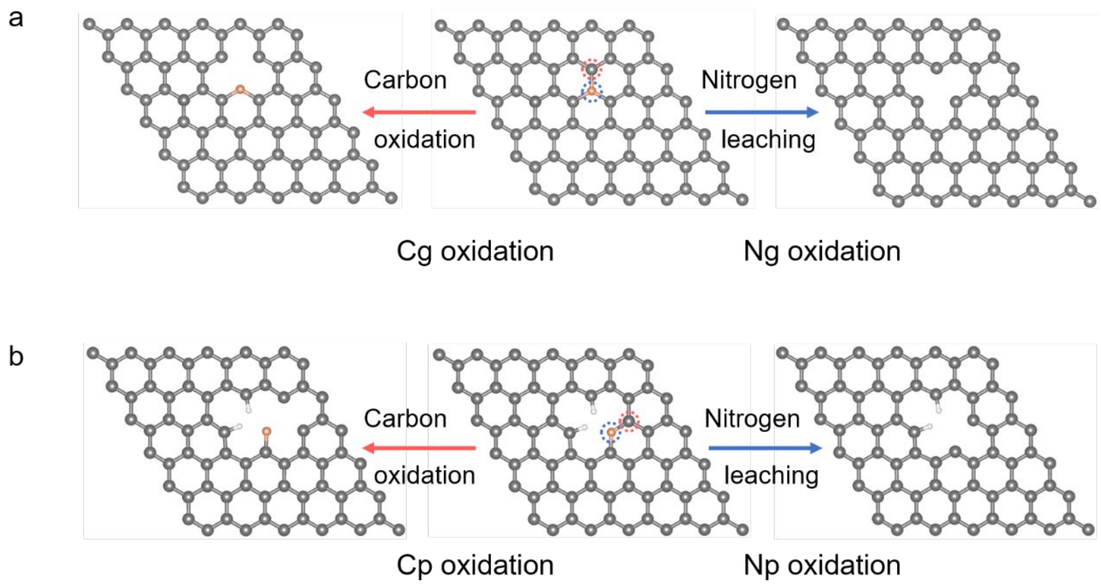

Figure S14. The top views of the optimized geometries of the DFT models before and after oxidation reaction.

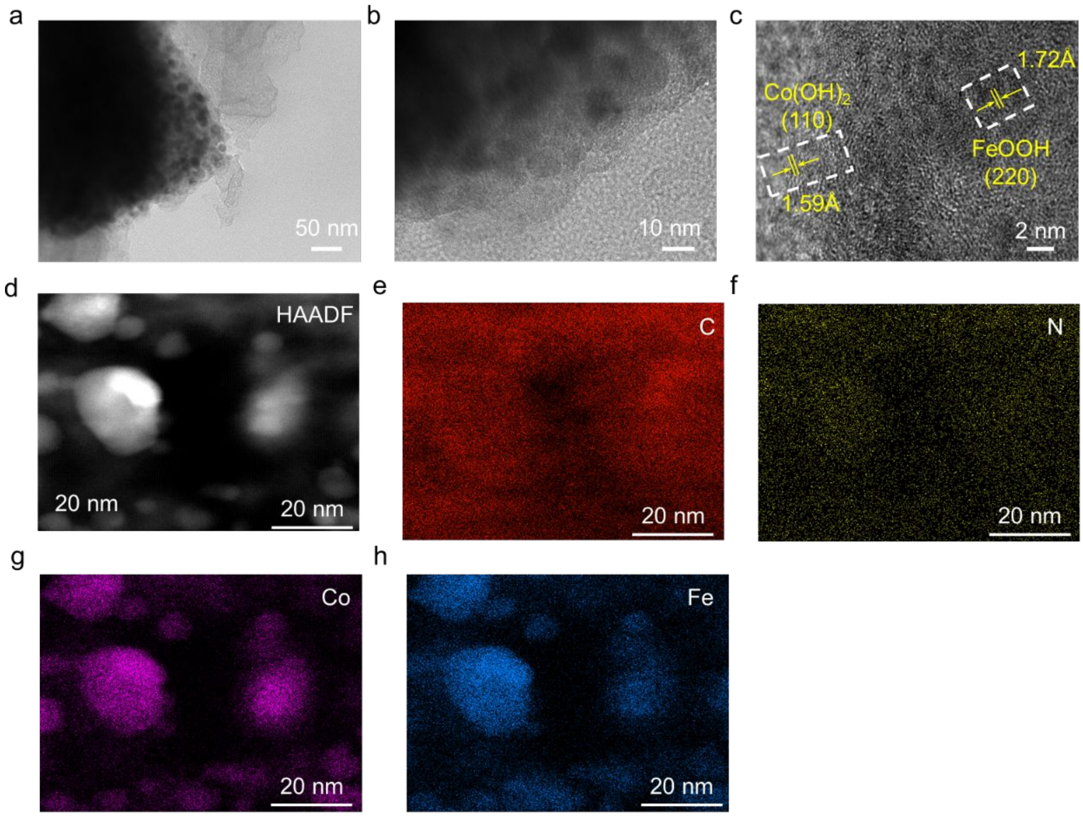

Figure S15. TEM images of $CFNC_{11.5}$. (a) ,(b) bright-filed micrographs with different magnifications; (c) HRTEM micrograph; (d)-(e) STEM-HAADF image and the corresponding EDX elemental mappings.

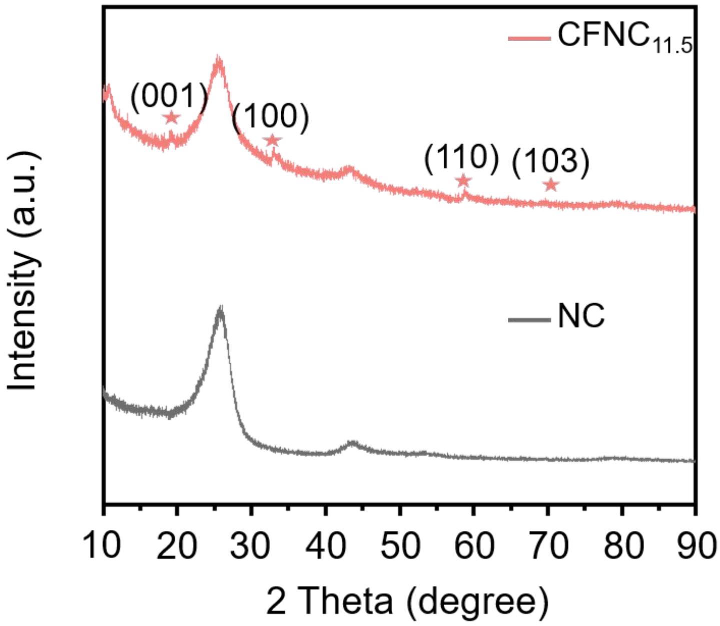

Figure S16. The XRD patterns of NC and $CFNC_{11.5}$.

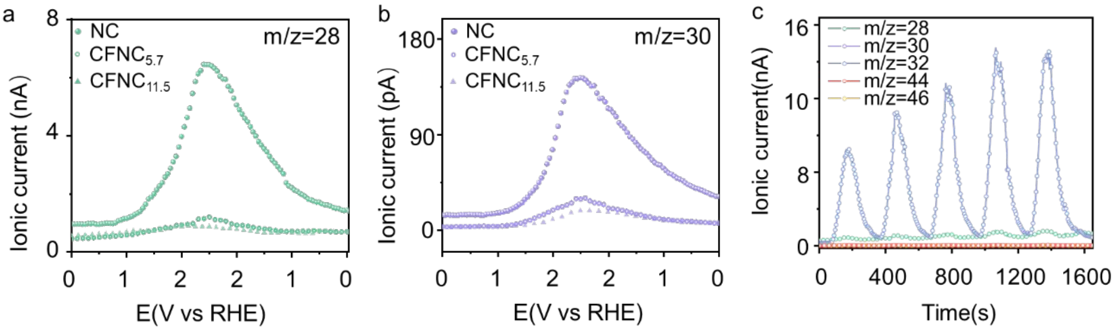

Figure S17. (a,b) the DEMS signals at m/z=28 and 30 after depositing different mass loadings of $CoFe(OH)_x$ in one CV cycle; d) the comparison of five signal (m/z= 28, 30, 32, 44, 46) of $CFNC_{11.5}$ in five CV cycles.

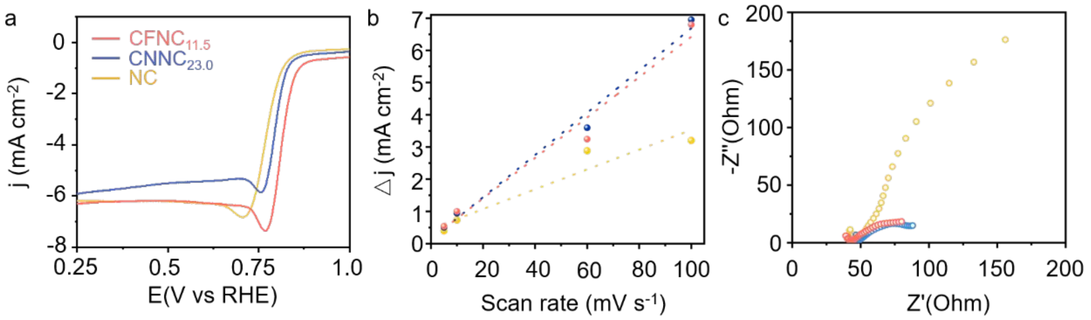

Figure S18. (a)The ORR LSV curves at 1600 rpm; (b) the double layer capacitance lots and (c) EIS spectra (at $E_{1/2}$) of CFNC with different mass loading.

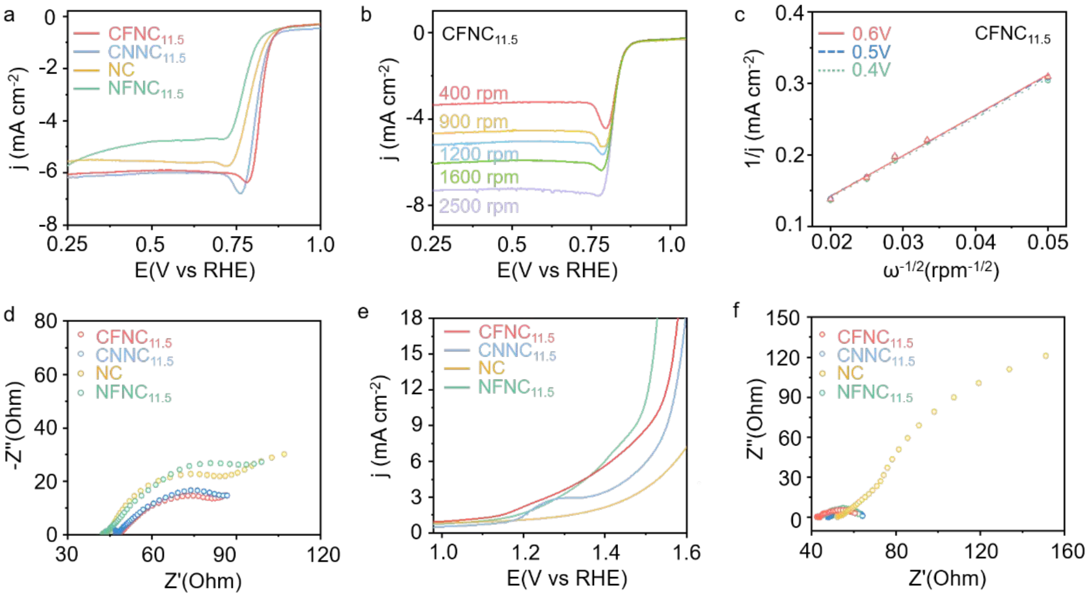

Figure S19. (a) ORR LSVs of various catalysts at 1600 rpm; (b) LSVs at different rotating speeds of $CFNC_{11.5}$; (c) Koutecky–Levich plot and (d) EIS spectra at $E_{1/2}$. (e) OER LSVs of various catalysts, (f) EIS spectra at E_{10}. The scan rate is 5 mV s^{-1}, with iR compensation.

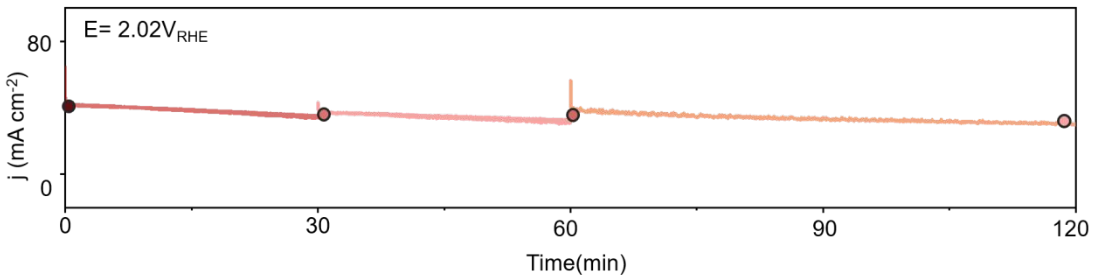

Figure S20. The i-t curve of $CFNC_{11.5}$ at 2.02V vs. RHE for 120min.

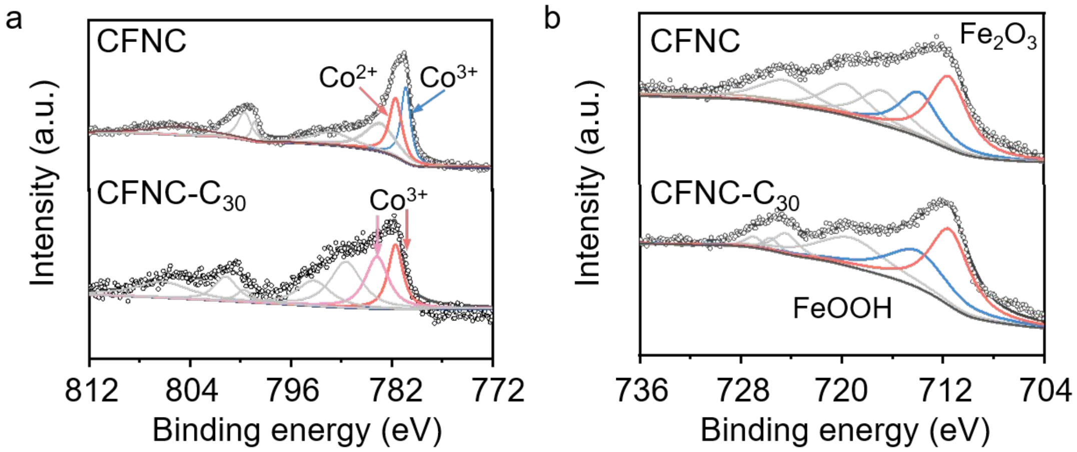

Figure S21. The XPS spectra comparison of CFNC and CFNC-C_{30} at (a) Co2p and (b) Fe2p core-level.

Table S1. Summary of atomic ratios of NC and NC-O_{100} from XPS data.

Samples	Carbon	Nitrogen	Oxygen
NC	85.26%	7.36%	7.38%
NC-O_{100}	69.36%	1.70 %	28.94%

Table S2. The ORR performance of NC and hybrid catalysts coupling various MOH.

Samples	$E_{1/2}$
NC	0.79
$CFNC_{11.5}$	0.828
$NFNC_{11.5}$	0.816
$CNNC_{11.5}$	0.778
Co-NC	0.811
Fe-NC	0.802
Ni-NC	0.785
